# *Petrocodon dahuaensis* (Gesneriaceae), a new species from the limestone area of Guangxi, China

**DOI:** 10.3897/phytokeys.272.181552

**Published:** 2026-03-31

**Authors:** Ming-Lin Mo, Shi-Li Chang, You-Dong Wu, Yi Zhang, Zhao-Cen Lu, Chen Feng

**Affiliations:** 1 Guangxi Key Laboratory of Plant Conservation and Restoration Ecology in Karst Terrain, Guangxi Institute of Botany, Guangxi Zhuang Autonomous Region and Chinese Academy of Sciences, Guilin, 541006, Guangxi, China Guangxi Key Laboratory of Plant Conservation and Restoration Ecology in Karst Terrain, Guangxi Institute of Botany, Guangxi Zhuang Autonomous Region and Chinese Academy of Sciences Guilin China; 2 College of Life Sciences, Guangxi Normal University, Guilin, 541006, Guangxi, China College of Life Sciences, Guangxi Normal University Guilin China; 3 Jiangxi Provincial Key Laboratory of Ex Situ Plant Conservation and Utilization, Lushan Botanical Garden, Chinese Academy of Sciences, Jiujiang, 332900, Jiangxi, China Jiangxi Provincial Key Laboratory of Ex Situ Plant Conservation and Utilization, Lushan Botanical Garden, Chinese Academy of Sciences Jiujiang China

**Keywords:** Morphology, new taxon, *Petrocodon asterostriatus*, taxonomy

## Abstract

*Petrocodon dahuaensis*, a new species from a limestone area of Guangxi, China, is described and illustrated. The new species is similar to *P. asterostriatus* but differs in its leaf blade, cordate to subrounded, margin crenate to serrate; bracts, bracteoles, and calyx smaller; corolla purple, base straight to slightly inflated; filaments purple; and style densely glandular-puberulent. Molecular evidence also supports its close relationship with *P. asterostriatus*. According to the IUCN Red List Categories and Criteria, the provisional conservation status of *P. dahuaensis* is assessed as Data Deficient (DD).

## Introduction

The genus *Petrocodon* Hance was an oligotypic genus for over a century (Wang et al. 1998), with only three species and one variety recorded (Wei 2007; Jiang et al. 2011). In 2011, *Petrocodon* was redefined based on molecular and morphological evidence and was expanded to include the following elements: the genera *Calcareoboea* C.Y.Wu ex H.W.Li, *Dolicholoma* D.Fang & W.T.Wang, *Paralagarosolen* Y.G.Wei, *Tengia* Chun, and *Lagarosolen* W.T.Wang; along with the species *Wentsaiboea tiandengensis* Yan Liu & B.Pan and *Primulina guangxiensis* Yan Liu & W.B.Xu (Wang et al. 2011; Weber et al. 2011). This revision greatly expanded the morphological diversity of *Petrocodon*, particularly in flower morphology. Recently, according to phylogenetic reconstructions, three species were transferred into *Petrocodon* from *Allocheilos* W.T.Wang (Liu et al. 2024). Currently, the genus *Petrocodon* comprises 61 species and one variety (GRC 2025; Liu et al. 2025; Shi et al. 2025; Wei et al. 2025) and is mainly distributed in southern to southwestern China, with a few species occurring in northern Vietnam, Thailand, and central China (Huang et al. 2023). The Sino–Vietnam border region, especially the limestone area of Guangxi, is the diversification center of the genus *Petrocodon*, hosting 31 species (Li et al. 2025; Liu et al. 2025; Shi et al. 2025; Tan et al. 2025; Wei et al. 2025).

During the investigation of plant diversity from March 2023 to July 2024, we collected an unknown flowering plant of Gesneriaceae. It was growing on moist rock surfaces at the entrance of a karst cave in Dahua County, Guangxi, China. Based on its lithophytic habit and the morphological characteristics of its cymes, flowers, and leaf blades, we tentatively identified it as a member of *Petrocodon*. After carefully checking the flowering plant, specimens, and relevant literature (Fang et al. 1993; Wei et al. 2008; Xu et al. 2010; Chen et al. 2014; Li et al. 2020; Yang et al. 2022; Li et al. 2025) and performing molecular phylogenetic analyses, we found that it did not match any known taxa of *Petrocodon* and confirmed that it is a new species.

## Materials and methods

### Taxon sampling and morphological comparison

Specimens of this new species were collected from Dahua County, Guangxi, China. Fresh flowers and dry fruits from several individuals were randomly selected, meticulously observed, and photographed. Morphological measurements of rhizomes, leaf blades, cymes, flowers, and fruits were taken from both specimens and photographs. Then, we carefully examined specimens of *Petrocodon* (including types) deposited in CSFI, GXMG, IBK, IBSC, and PE (herbarium codes follow Thiers 2025). We also compared other species of *Petrocodon* using online images from the Chinese Virtual Herbarium (https://www.cvh.ac.cn/), GCCC (https://gccc.818time.cf), and JSTOR Global Plants (https://plants.jstor.org/).

### Phylogenetic analysis

**Figure 1.**
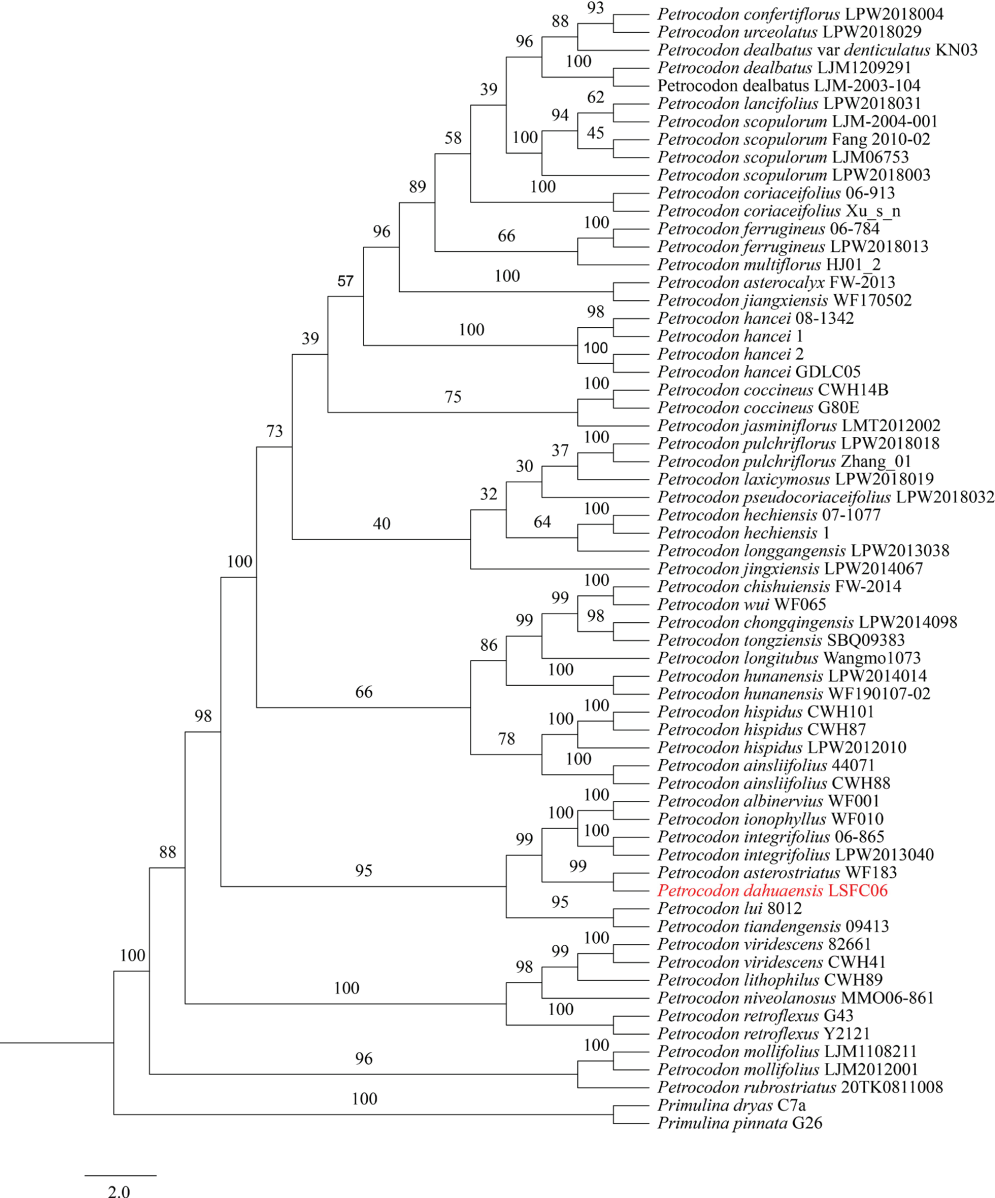
Phylogenetic tree of *Petrocodon* generated from maximum likelihood (ML) of *trnL-F* and ITS datasets. Numbers on the branches indicate ML bootstrap values (≥ 30%).

Leaf material of this new species was collected from Dahua County and dried immediately in silica gel for DNA extraction (Doyle and Doyle 1987). We sequenced the nuclear ribosomal internal transcribed spacer (ITS) region and the plastid *trnL-F* region. Primers, DNA extraction, PCR amplification, and sequencing followed Yang et al. (2022). To explore the phylogenetic relationships of the undescribed species and other species within the genus, we incorporated 62 samples from 42 species (Table 1), following Zhang et al. (2023). The ingroup comprised 60 samples from 40 species of *Petrocodon*. *Primulina pinnata* (W.T.Wang) Yin Z.Wang and *P. dryas* (Dunn) Mich.Möller & A.Weber were selected as outgroups based on previous phylogenetic studies (Möller et al. 2009; Weber et al. 2011; Zhang et al. 2023). Phylogenetic analysis was conducted using a combined *trnL-F* and ITS dataset with Maximum Likelihood (ML) in IQ-TREE v.2.0.6 (Nguyen et al. 2015), employing 1000 bootstrap replicates and ModelFinder (Kalyaanamoorthy et al. 2017), which identified K81+R2 as the best-fit substitution model. The tree was visualized using FigTree v.1.4.3 (http://tree.bio.ed.ac.uk/software/figtree/). Topologies of the *trnL-F* and ITS datasets were visually compared, and no significant inconsistencies were observed at nodes with bootstrap support ≥ 80% (results not shown) (Fu et al. 2022). Consequently, the two regions were combined in a total evidence analysis (Fig. 1).

**Table 1.** The voucher and GenBank accession numbers used in this study.

Species name	Sample	Voucher	*trnL-F*	ITS
*Primulina dryas*	C7a	T.C.Godfrey 369 (E)	FJ501524	FJ501348
*Primulina pinnata*	G26	Expedition Beijing 896526 (US)	FJ501526	FJ501349
*Petrocodon ainsliifolius*	44071	Y.M.Shui et al. 44071 (KUN)	HQ632941	HQ633038
	CWH88	Y.M.Shui et al. 44071/B (KUN)	KF202298	KF202291
*Petrocodon albinervius*	WF001	WF001	ON959495	ON950050
*Petrocodon asterocalyx*	FW-2013	–	KC904957	KC904954
*Petrocodon asterostriatus*	WF183	WF183	ON959497	ON950052
*Petrocodon chishuiensis*	FW-2014	–	KF680503	KF680504
*Petrocodon chongqingensis*	LPW2014098	P.W.Li LPW2014098 (PE)	MN637569	MN627918
*Petrocodon coccineus*	CWH14B	GBOWS 290	KF202299	KF202292
	G80E	MMO 01-141	FJ501516	FJ501341
*Petrocodon confertiflorus*	LPW2018004	P.W.Li LPW2018004 (PE)	MN637556	MN627906
*Petrocodon coriaceifolius*	06-913	M.Moeller MMO 06-913 (E)	HQ632943	HQ633040
	Xu_s_n	W.B.Xu s.n. (IBK)	KY796060	KY796058
***Petrocodon dahuaensis***	**LSFC06**	**Xu et al. 16041 (IBK)**	**PP898342**	**PP906178**
*Petrocodon dealbatus*	LJM1209291	LJM1209291 (HEAC)	KR476565	KR337020
	KN03	Y.G. Wei 2010-03 (IBK)	JF697590	JF697578
	LJM-2003-104	LJM-2003-104 (PE)	GU350668	GU350636
*Petrocodon ferrugineus*	06-784	M.Moeller MMO 06-784 (E)	HQ632946	HQ633043
	LPW2018013	P.W.Li LPW2018013 (PE)	MN637550	MN627900
*Petrocodon hancei*	08-1342	M.Moeller MMO 08-1342 (E)	HQ632944	HQ633041
	1	–	KC904959	KC904956
	2	–	KC904958	KC904955
	GDLC05	–	KF498253	KF498051
*Petrocodon hechiensis*	1	–	KR476563	KR337018
	07-1077	M.Moeller MMO 07-1077 (E)	HQ632942	HQ633039
*Petrocodon hispidus*	CWH101	Y.M.Shui et al. B2012-082/B	KF202301	KF202294
	CWH87	Y.M.Shui et al. B2012-082 (KUN)	KF202300	KF202293
	LPW2012010	P.W.Li LPW2012010 (PE)	MN637566	MN627915
*Petrocodon hunanensis*	WF190107-02	WF190107-02	MK941180	MK941179
	LPW2014014	P.W.Li LPW2014014 (PE)	MN637570	MN627919
*Petrocodon integrifolius*	06-865	M.Moeller MMO 06-865	HQ632940	HQ633037
	LPW2013040	P.W.Li LPW2013040 (PE)	MN637571	MN627920
*Petrocodon ionophyllus*	WF010	WF010	ON959496	ON950051
*Petrocodon jasminiflorus*	LMT2012002	M.T.Liu LMT2012002	MN637568	MN627917
*Petrocodon jiangxiensis*	WF170502	WF170502	MH699398	MH699397
*Petrocodon jingxiensis*	LPW2014067	P.W.Li LPW2014067 (PE)	MN637544	MN627894
*Petrocodon lancifolius*	LPW2018031	M.Q.Han LPW2018031	MN637552	MN627902
*Petrocodon laxicymosus*	LPW2018019	P.W.Li LPW2018019	MN637549	MN627899
*Petrocodon lithophilus*	CWH89	Y.M.Shui et al. B2012-078	KF202302	KF202295
*Petrocodon longgangensis*	LPW2013038	P.W.Li LPW2013038 (PE)	MN637546	MN627896
*Petrocodon longitubus*	Wangmo 1073	C.R.Li Wangmo1073	MN637543	MN627893
*Petrocodon lui*	8012	Y.G.Wei 8012 (IBK)	HQ632938	HQ633035
*Petrocodon mollifolius*	LJM2012001	J.M.Li LJM2012001	MN637539	MN627889
	LJM1108211	LJM1108211 (HEAC)	KR476547	KR337000
*Petrocodon multiflorus*	HJ01-2	–	KM232660	KJ475411
*Petrocodon niveolanosus*	MMO06-861	M.Moeller MMO06-861 (E)	JF697588	JF697576
*Petrocodon pseudocoriaceifolius*	LPW2018032	P.W.Li LPW2018032	MN637547	MN627897
*Petrocodon pulchriflorus*	LPW2018018	P.W.Li LPW2018018	MN637565	MN627914
	Zhang_01	Q.Zhang 01 (IBK)	KX579059	KX579058
*Petrocodon retroflexus*	Y2121	Y2121 (IBK)	KX579061	KX579060
	G43	Y.S.Huang G43	MN637540	MN627890
*Petrocodon rubrostriatus*	20TK0811008	20TK0811008	OQ955755	OQ968811
*Petrocodon scopulorum*	LPW2018003	M.T.Liu LPW2018003	MN637562	MN627911
	Fang 2010-02	W.Fang 2010-02 (IBK)	HQ632947	HQ633044
	LJM-2004-001	LJM-2004-001 (PE)	GU350669	GU350637
	LJM06753	LJM06753 (PE)	KR476567	KR337023
*Petrocodon tiandengensis*	09413	W.B.Xu s.n. (IBK)	JX506850	JX506960
*Petrocodon tongziensis*	SBQ09383	–	MF872618	MF872617
*Petrocodon urcelotatus*	LPW2018029	P.W.Li LPW2018029	MN637559	MN627909
*Petrocodon viridescens*	82661	Y.M.Shui et al. 82661 (E)	HQ632939	HQ633036
	CWH41	Y.M.Shui et al. 85339	KF202304	KF202297
*Petrocodon wui*	WF065	WF065	OQ716553	OQ694978

Note. “–” indicates that the author did not provide the voucher number.

## Results and discussion

**Figure 2.**
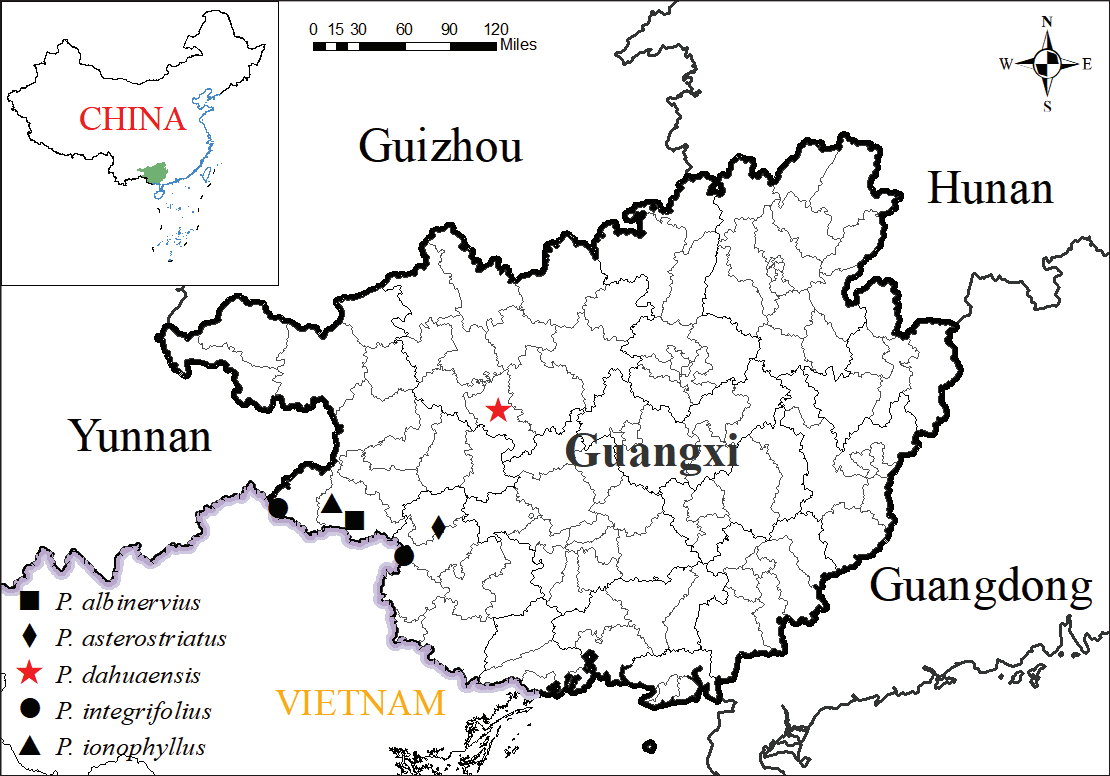
Distribution map of *Petrocodon dahuaensis* and its allies.

As in previous studies (Chen et al. 2014; Shi et al. 2024), all sampled *Petrocodon* taxa clustered together as a monophyletic group (BS = 100%), but the phylogenetic relationships within *Petrocodon* were poorly resolved, with many nodes having bootstrap support below 70%. However, our analyses of molecular data show that *P. dahuaensis* is placed within a strongly supported clade (BS = 99%), which also includes *P. albinervius* D.X.Nong & Y.S.Huang (Nong et al. 2021), *P. asterostriatus* F.Wen, Y.G.Wei & W.C.Chou (Yang et al. 2022), *P. ionophyllus* F.Wen, S.Li & B.Pan (Li et al. 2020), and *P. integrifolius* (D.Fang & L.Zeng) A.Weber & Mich.Möller (Fang et al. 1993) (Fig. 1). The five species in this clade share broadly ovate, cordate to subrounded leaf blades, purple corollas with internal longitudinal stripes, and triangular to narrowly triangular corolla lip lobes. They are all endemic to limestone regions in Guangxi and occupy similar habitats. However, the other four species are endemic to the Sino–Vietnam border region, while *P. dahuaensis* is distributed in Dahua County, Guangxi (Fig. 2).

Within this clade, the new species is most closely related to *P. asterostriatus* (BS = 99%) (Fig. 1), with which it also shares morphological similarities but differs in its leaf blade, cordate to subrounded, margin crenate to serrate; bracts, bracteoles, and calyx smaller; corolla purple, base straight to slightly inflated; filaments purple; and style densely glandular-puberulent.

Based on phylogenetic and morphological evidence, we confirm that it is a species new to science and describe the new species as *Petrocodon dahuaensis* M.L.Mo, Chen Feng & Z.C.Lu. Detailed morphological differences between *P. dahuaensis* and its allies are shown in Table 2.

**Table 2.** Morphological comparison between *Petrocodon dahuaensis* and its allies.

Characters	*P. dahuaensis*	*P. asterostriatus*	*P. integrifolius*	*P. albinervius*	*P. ionophyllus*
Leaf blade	cordate to subrounded, margin crenate or serrate	broadly ovate to subcordate, margin entire	ovate, broadly ovate to orbicular, margin entire	broadly ovate to suborbicular, margin entire	ovate or broadly ovate, margin entire
Petiole	6–20 cm long	6–13 cm long	2–8.5 cm long	1.5–4 cm long	1.2–1.5 cm long
Lateral veins	4–5	4–7	4–7	4–6	2–4
Bracts	ovate to ovate-lanceolate, 4–9 mm long	broadly lanceolate or ovate, 10–25 mm long	oblong to lanceolate, 3–8 mm long	elliptic, 8–10 mm long	lanceolate, 6–10 mm long
Bracteoles	elliptic, ca. 2 mm long	linear-lanceolate, 8–9 mm long	usually lacking, 1.5–2 mm long	lanceolate, 4–5 mm long	not seen
Calyx lobe	lanceolate to linear-lanceolate, 5–7 mm long	lanceolate to linear-lanceolate, 8–16 mm long	narrowly triangular, 7–11 mm long	narrowly lanceolate, 5–6 mm long	lanceolate to linear-lanceolate, 6–8 mm long
Corolla tube	purple, broadly funnelform	pale purplish-maroon, infundibuliform	purple, thin cylindrical	white to pale purple, funnelform	purple, infundibuliform
Corolla base	straight to slightly inflated	near spherical	straight	slightly inflated	straight to slightly inflated
Filaments	purple, distally covered with glandular puberulence	white, distally covered with dark purple glands	white, pubescent, and glandular-puberulent	white, glabrous	white, glandular-puberulent
Lateral staminodes	ca. 1.5 mm long	ca. 5 mm long	ca. 1.5 mm long	3–5 mm long	9–10 mm long
Pistil	ca. 1.6 cm long	ca. 2.2 cm long	ca. 1.6 cm long	2–2.5 cm long	1.2–1.3 cm long
Style	densely glandular-puberulent	densely puberulent	glandular-puberulent and pubescent	pubescent	densely glandular-puberulent

### Taxonomic treatment

#### Petrocodon dahuaensis

Taxon classificationPlantaeLamialesGesneriaceae

M.L.Mo, Chen Feng & Z.C.Lu sp. nov.

9B10FF69-9670-5F94-9F53-4281D9DEDC5E

urn:lsid:ipni.org:names:77378241-1

Figs 3, 4

##### Chinese name.

dà huà shí shān jù tái (大化石山苣苔).

**Figure 3.**
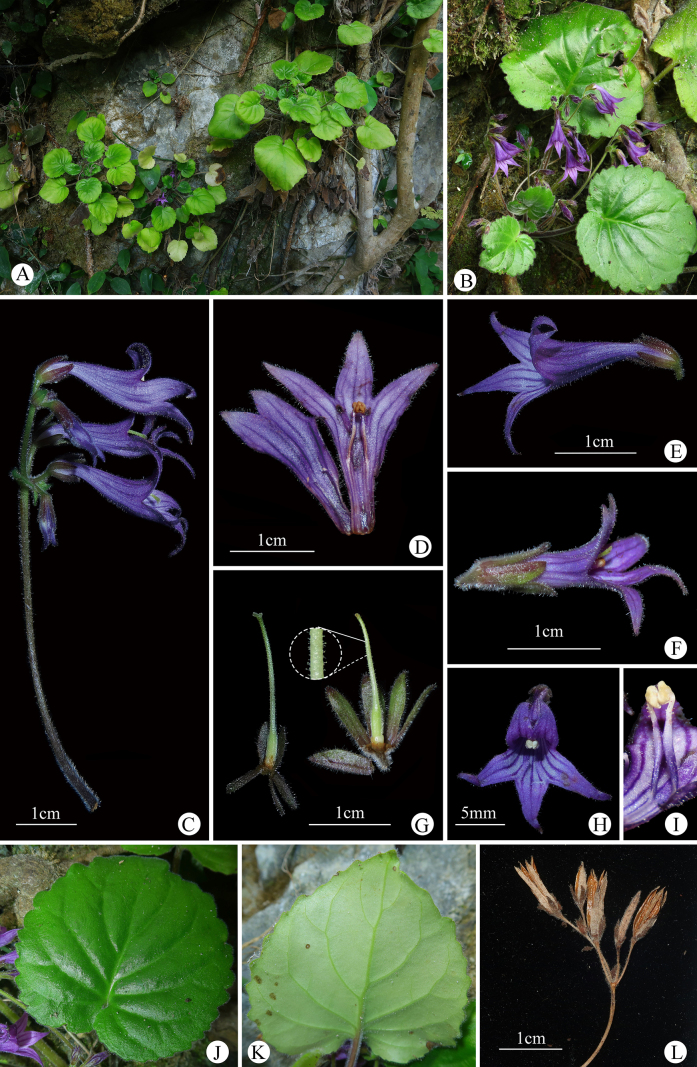
*Petrocodon dahuaensis* sp. nov. **A**. Habit; **B**. Flowering plant; **C**. Cyme and bracts; **D**. Opened corolla; **E**. Lateral view of flower; **F**. Back view of flower; **G**. Pistils and calyces; **H**. Frontal view of flower; **I**. Stamens; **J**. Adaxial view of leaf blade; **K**. Abaxial view of leaf blade; **L**. Capsules.

**Figure 4.**
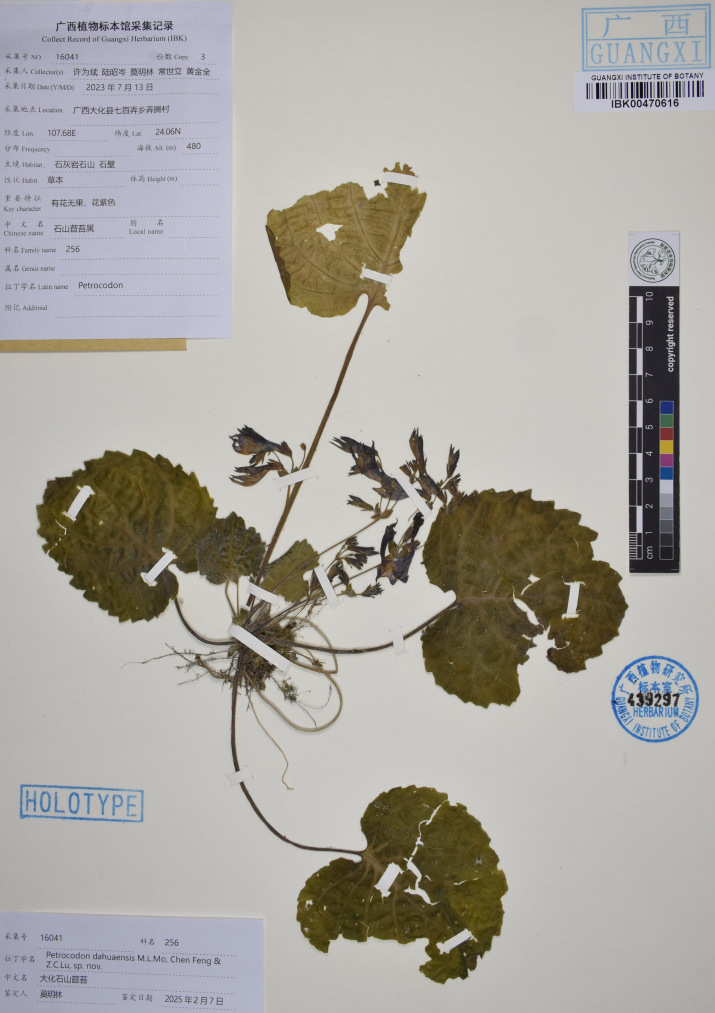
The holotype sheet of *Petrocodon dahuaensis* (IBK).

##### Diagnostic.

The new species is similar to *Petrocodon asterostriatus* F.Wen, Y.G.Wei & W.C.Chou, but differs by its leaf blades cordate to subrounded, margin crenate or serrate (vs. broadly ovate to subcordate, margin entire), bracts 4–9 mm long (vs. 10–25 mm long), bracteoles ca. 2 mm long (vs. 9–18 mm long), calyx 5–7 mm long (vs. 8–16 mm long), corolla purple (vs. pale purplish-maroon), base straight to slightly inflated (vs. near spherical), filaments purple (vs. white), and style densely glandular-puberulent (vs. densely puberulent).

##### Type.

China • Guangxi: Hechi City, Dahua County, Qibainong Township, Nongteng Village, 24.06°N, 107.68°E, growing on moist rock surfaces at the entrance of a karst cave, alt. 480 m, 13 July 2023, *Xu et al. 16041* (holotype: IBK00470616, isotypes: IBSC and PE).

##### Description.

Perennial herb. Rhizome erect or ascending, 1–5 cm long, 7–15 mm in diam. Leaves 8–18, basal. Petiole green to reddish brown, densely pubescent, 6–20 cm long. Leaf blade papery, cordate to subrounded, margin crenate or serrate, 4–10 × 4–12 cm, apex obtuse, base cordate; adaxial side deeply green, pubescent; abaxial side pale green, pubescent, densely pubescent along the veins; lateral veins 4–5 on each side of the midrib. Cymes 4–8, axillary, 1–2-branched, 4–10-flowered or more; peduncle green to reddish brown, 3.5–11.6 cm long, densely pubescent; bracts 2, opposite, green with reddish brown at base, ovate to ovate-lanceolate, apex acute, margin entire, 4–9 × 1.2–2.5 mm, both surfaces densely pubescent; bracteoles 2, opposite, elliptic, ca. 2 × 0.5 mm, color and indumentum similar to bracts. Pedicel green, 4–6 mm long, ca. 1 mm in diam., densely pubescent. Calyx 5-parted to base, green to reddish brown, lanceolate to linear-lanceolate, 5–7 × 1.2–2.1 mm, densely pubescent on both surfaces. Corolla purple, zygomorphic, 3–3.5 cm long, base straight to slightly inflated, outside puberulent and sparsely glandular-puberulent, inside glabrous with deep purple longitudinal stripes; tube infundibuliform, ca. 17 mm long, ca. 3 mm in diam. at base, ca. 7 mm in diam. at tube opening; limb 2-lipped; adaxial lip 2-lobed, triangular to narrowly triangular, lobes ca. 5 mm long, ca. 4 mm wide at base, margin entire, apex acuminate; abaxial lip 3-lobed to more than middle, narrowly lanceolate to narrowly triangular, lobes ca. 9 mm long, 4–5 mm wide at base, margin entire, apex acuminate. Stamens 2, adnate to 8–10 mm above corolla tube base; filaments straight, purple, 5–6 mm long, sparsely puberulent from the base to above middle, distally with sparse glandular puberulence; anthers pale brownish yellow, reniform, ca. 1.2 mm long, glabrous, coherent face to face. Staminodes 3, glabrous, adnate to 7–9 mm above corolla tube base, lateral ones ca. 1.5 mm long, middle one ca. 1 mm long. Disc annular, ca. 1 mm high, margin entire. Pistil ca. 1.6 cm long; ovary cylindrical, 4.5 mm long, 1–1.4 mm in diam., densely pubescent; style ca. 12 mm long, ca. 0.6 mm in diam., densely glandular-puberulent; stigma bilobed, lobes ovate, ca. 0.5 mm long. Capsules linear, straight, 0.9–1.1 cm long, densely puberulent, 4-valved.

##### Etymology.

The specific epithet “*dahuaensis*” refers to the type locality of this new species.

##### Phenology.

Flowering from June to July.

##### Distribution and habitat.

*Petrocodon dahuaensis* is endemic in Guangxi, China, and is currently known only from the type locality. It grows on shaded and moist rock surfaces at the northeast-facing entrance of karst caves, at an altitude of about 500 m. The new species is dominant in the herbaceous layer of this small habitat. Associated plants include *Begonia* sp., *Boehmeria nivea* (L.) Gaudich., *Elatostema* sp., *Iodes cirrhosa* Turcz., *Pothos chinensis* (Raf.) Merr., *Selaginella uncinata* (Desv.) Spring, and *Zenia insignis* Chun, among others.

##### Additional specimens examined (paratypes).

China • Guangxi: Hechi City, Dahua County, Qibainong Township, Nongteng Village, 24.06°N, 107.68°E, growing on moist rock surfaces at the entrance of a karst cave, alt. 480 m, 11 March 2023, Xu et al. *15539* (IBK).

##### Provisional conservation status.

Currently, *Petrocodon dahuaensis* is known only from the type locality, with a total number of individuals less than 50. As further surveys across its potential distribution are lacking, the existence of other populations remains unclear. Therefore, *P. dahuaensis* is provisionally classified as Data Deficient (DD), according to the IUCN Red List Categories and Criteria (IUCN 2022).

## Supplementary Material

XML Treatment for Petrocodon dahuaensis

## Additional information

### Use of AI

No use of AI was reported.

### Author ORCIDs

Ming-Lin Mo https://orcid.org/0009-0000-8895-5474

Shi-Li Chang https://orcid.org/0009-0005-9801-4121

You-Dong Wu https://orcid.org/0009-0006-6650-027X

Yi Zhang https://orcid.org/0000-0003-2871-8033

Zhao-Cen Lu https://orcid.org/0000-0002-1725-0247

Chen Feng https://orcid.org/0000-0003-1144-0047

### Data availability

All of the data that support the findings of this study are available in the main text.
